# Bi-fluorescent *Staphylococcus aureus* infection enables single-cell analysis of intracellular killing *in vivo*

**DOI:** 10.3389/fimmu.2023.1089111

**Published:** 2023-01-23

**Authors:** Kristina D. Hinman, Sonia S. Laforce-Nesbitt, Joshua T. Cohen, Miles Mundy, Joseph M. Bliss, Alexander R. Horswill, Craig T. Lefort

**Affiliations:** ^1^ Division of Surgical Research, Department of Surgery, Rhode Island Hospital, Providence, RI, United States; ^2^ Warren Alpert Medical School, Brown University, Providence, RI, United States; ^3^ Graduate Program in Pathobiology, Brown University, Providence, RI, United States; ^4^ Department of Pediatrics, Women and Infants Hospital, Providence, RI, United States; ^5^ Department of Immunology and Microbiology, University of Colorado School of Medicine, Aurora, CO, United States

**Keywords:** infection, Staphylococcus aureus, phagocytosis, reactive oxygen species, neutrophil, flow cytometry

## Abstract

Techniques for studying the clearance of bacterial infections are critical for advances in understanding disease states, immune cell effector functions, and novel antimicrobial therapeutics. Intracellular killing of *Staphylococcus aureus* by neutrophils can be monitored using a *S. aureus* strain stably expressing GFP, a fluorophore that is quenched when exposed to the reactive oxygen species (ROS) present in the phagolysosome. Here, we expand upon this method by developing a bi-fluorescent *S. aureus* killing assay for use *in vivo.* Conjugating *S. aureus* with a stable secondary fluorescent marker enables the separation of infected cell samples into three populations: cells that have not engaged in phagocytosis, cells that have engulfed and killed *S. aureus*, and cells that have viable internalized *S. aureus*. We identified ATTO647N-NHS Ester as a favorable dye conjugate for generating bi-fluorescent *S. aureus* due to its stability over time and invariant signal within the neutrophil phagolysosome. To resolve the *in vivo* utility of ATTO647N/GFP bi-fluorescent *S. aureus*, we evaluated neutrophil function in a murine model of chronic granulomatous disease (CGD) known to have impaired clearance of *S. aureus* infection. Analysis of bronchoalveolar lavage (BAL) from animals subjected to pulmonary infection with bi-fluorescent *S. aureus* demonstrated differences in neutrophil antimicrobial function consistent with the established phenotype of CGD.

## Introduction

The inflammatory response to infection is a dynamic process with tissue-specific variability: leukocytes are recruited to cross endothelial barriers at different times, with specificity dependent on the cytokine environment and cellular affinity for endothelial adhesion receptors (1, 2). In response to the initial insult, resident cells such as macrophages or natural killer cells recruit innate immune cells as the first line of defense (3–5). In the acute phase, neutrophils are the most abundant infiltrating leukocyte in response to bacterial infections. This recruitment is self-amplified by neutrophil-derived signals such as interleukin-8 (IL-8) and leukotriene B4 (LTB4) (3, 6). Tight regulation over recruitment is crucial as neutrophil depletion is often fatal while overactivation leads to enzymatic and oxidative damage to the tissues involved (4, 7).

Neutrophils clear bacterial infections through numerous intra- and extracellular effector functions, depending on the species of bacteria. *Staphylococcus aureus* is one species of interest due to its prevalence, ability to evade the immune system, and increasing resistance to pharmaceutical anti-microbial agents. As of 2018, methicillin-resistant *S. aureus* (MRSA) was the leading cause of death resulting from an antibiotic-resistant pathogen (8). Neutrophils predominantly kill *S. aureus via* internalization into a phagolysosome (5). Within the phagolysosome, the nicotinamide adenine dinucleotide phosphate (NADPH) oxidase complex generates reactive oxygen species (ROS) *via* a series of reactions referred to as the oxidative burst (7, 9). The proposed mechanisms of bacterial killing by ROS include oxidative damage of the microbe membrane, DNA, and other cellular components (7, 10, 11). In addition, NADPH oxidase activity may modulate the potency of proteolytic enzymes that are delivered to the phagosome and contribute to microbicidal activities (10, 12).

Numerous pathologies affect the neutrophil-dependent clearance of bacteria. An impaired neutrophilic response may be the result of depleted cell counts (neutropenia), the inability to recruit cells to inflamed tissue sites, or loss of effector functions (13). Neutrophil dysfunction can be caused by a primary immunodeficiency or can occur secondary to a disease state, such as sepsis or cancer (5, 9, 14). There are sufficient experimental methods for quantifying neutrophil counts to evaluate defects in production or tissue-specific recruitment, including the characterization of surface markers by flow cytometry or morphology by cytospin. However, methods for evaluating microbe killing on a single cell level *in vivo* are relatively limited. Current methods depend on comparing pathogen clearance through plating tissue homogenates and counting colony forming units (CFU) *ex vivo*. This method is limited as it is non-specific, only providing insight into overall host defense discrepancies without identifying the specific effector cell(s) responsible for observed differences. *In vitro* experiments may implicate cellular function more specifically but exclude the influence of host extracellular factors and proteins that aid in the clearance of *S. aureus* (15). Alternatives based on live *in vivo* imaging require advanced technology which is expensive, time-consuming, and limited by the target tissue location (16). Therefore, we sought to develop a fluorescence-based assay to quantify *in vivo* neutrophil antimicrobial function on a single cell level.

Previous work by Schwartz, et al. has demonstrated *in vitro* that the fluorescent signal from a GFP-producing *S. aureus* is quenched in a ROS-dependent manner over time (17, 18). This loss of fluorescence is correlated with intracellular *S. aureus* viability. The mechanism of GFP bleaching is attributed to the HOCl in the phagosome, likely *via* chlorination of the tyrosine 66 residue in GFP (19). For a single point in time, the GFP signal alone is insufficient to distinguish cells that have not undergone phagocytosis of *S. aureus* from cells that have bleached the GFP fluorescent signal in conjunction with eradicating internalized *S. aureus*. Therefore, we screened for a secondary fluorescent dye capable of maintaining a robust fluorescent signal over time, independent of ROS production, phagolysosome activity, or *S. aureus* viability. We generated bi-fluorescent *S. aureus* by conjugating a far-red fluorescent dye to a GFP-producing USA300 *S. aureus*. In our study, we extensively characterize neutrophil intracellular killing of bi-fluorescent *S. aureus* to demonstrate the fidelity and utility of the assay for evaluating neutrophil antimicrobial function or dysfunction *in vivo*.

## Materials and methods

### *Staphylococcus aureus* culture and staining

Glycerol stocks of the superfolded GFP-producing USA300 strain of *S. aureus* were provided by Alexander Horswill and have been described (18). Liquid cultures were grown in tryptic soy broth (TSB, Sigma-Aldrich, St. Louis, MO) from the glycerol stock by incubating at 37C, shaking at 250 rpm, overnight. Before experimentation, overnight cultures were diluted and allowed to grow to the exponential phase. For staining, cultures were washed 2x in phosphate-buffered saline, pH=7.4 (PBS, Gibco, Dun Laoghaire, Co Dublin, Ireland), and brought to a concentration of 4x10^8^ CFU/mL. *S. aureus* was stained for 30 minutes in the dark at a final concentration of 0.5ug/mL of ATTO647N-NHS ester (Sigma-Aldrich). To pellet, cultures were centrifuged for 5 minutes at 3000 RPM. After staining, *S. aureus* was washed 2x in assay buffer (Hank’s balanced salt solution/10% fetal bovine serum/20 mM HEPES). Before use *in vitro*, *S. aureus* was opsonized by incubating at 37C and shaking for 5-10 minutes in assay buffer.

Growth curves were generated by bringing bi-fluorescent or GFP *S. aureus* to OD_600 =_ 0.1 in TSB. OD was measured using SmartSpec 3000 (Bio-Rad, Hercules, CA). Cultures were grown for four and a half hours, with a sample drawn every 30 minutes for the first 2 hours and then at 3 hours and 4.5 hours. At each time point, the OD_600_ was measured and the fluorescence of the *S. aureus* particles was measured using a MACSQuant Analyzer 10 flow cytometer (Miltenyi).

### HoxB8 conditionally immortalized progenitor derivation, culture,and differentiation

HoxB8 conditionally immortalized neutrophil progenitors were generated as previously described (20–22). Briefly, C57BL/6 mouse bone marrow was isolated, cultured, and transduced with a tamoxifen-inducible *Hoxb8* transgene. All cells were grown in Opti-Mem media supplemented with GlutaMax (Gibco), 30uM beta-mercaptoethanol (Sigma-Aldrich), 10% fetal bovine serum (Gemini Bio-Products, West Sacramento, CA), 1x penicillin/streptomycin (Gibco), 1x non-essential amino acids (Gibco) at a density of ~10^6^ cells/mL. The progenitor cell culture was supplemented with stem cell factor (SCF) and 100nM Z-4-hydroxytamoxifen (Tocris Bioscience, Bristol, UK) to induce *Hoxb8* expression. SCF was derived from the supernatant of CHO cells that secrete recombinant murine SCF (a gift from Patrice Dubreuil, Centre de Recherche en Cancérologie de Marseille). For differentiation, cells were washed 3x in PBS to remove Z-4-hydroxytamoxifen induction. Cells were pelleted by centrifugation at 400xg for 3 minutes. The differentiation culture was supplemented with SCF and 20 ng/mL G-CSF (BioLegend) for 2-3 days, and then just G-CSF until use on days 5-7.

### *In vitro* killing assay

Differentiated *Hoxb8* progenitor-derived neutrophils were suspended in assay buffer at a density of 1x10^7^ cells/mL. Samples treated with diphenyleneiodonium (DPI; Selleck Chemicals, Houston, TX) or cytochalasin D (Sigma-Aldrich) were incubated at 37°C for 20 minutes before infection. For each sample, one million cells were inoculated with 25µL of OD=0.5 *S. aureus* for an MOI of 20:1 for 15 minutes. Cells were then washed with warm lysostaphin 200ng/mL followed by assay buffer 1x. Cells were suspended in a final volume of 100µL of assay buffer. For killing assays, this was considered time zero. For experiments determining fluorescent intensity over time, every 20 minutes 10µL of the sample was plated in 200µL of cold PBS/1% FBS and kept on ice until analysis by the MACSQuant.

### Dead *S. aureus* phagocytosis assay

Bi-fluorescent *S. aureus* was heat-inactivated at 60C for 30 minutes. To evaluate the phagocytosis of dead *S. aureus*, cells were inoculated and washed using the same protocol as the live *S. aureus* killing assay. Cells were analyzed at time zero (after lysostaphin wash) to evaluate the internalization of the heat-killed *S. aureus.*

### Animal care and breeding

All studies were performed under the approval of the Lifespan Animal Welfare Committee (Protocol number 5017-19, Office of Laboratory Animal Welfare Assurance #A3922-01). These studies follow Public Health Service guidelines for animal care and use. The CGD mouse model was acquired from Jackson Laboratories (Bar Harbor, ME), strain B6.129S-Cybb^tm1Din^/J for in-house breeding. This strain was originally developed by knocking out the *Cybb* gene (gp91phox) to recapitulate the CGD phenotype. Female mice heterozygous for the knocked out *Cybb* gene (x-linked) were bred with wild-type males to yield hemizygous wild-type (WT) or knockout male progeny used in this study. Animals were housed in sterile caging until infection at 6-10 weeks old and provided access to water and standard chow *ad libitum*.

### *S. aureus* pulmonary infection

*S. aureus* was stained as previously described and brought to an OD_600 =_ 0.5 or 2x10^8^ CFU/mL. One mL of *S. aureus* was pelleted and suspended in 100µL of normal saline for a working solution. The inoculum was further diluted to provide a bolus of 6x10^7^CFU or 8x10^7^CFU in 40µL. Animals were anesthetized with vaporized isoflurane (Braintree Scientific, Braintree, MA) before oropharyngeal inhalation of the inoculum.

### *In vivo* killing assay

Twelve hours post pulmonary infection, mice were euthanized by Fatalplus (Vortech Pharmaceuticals, Dearborn, MI) and cervical dislocation. Bronchoalveolar lavage (BAL) was collected by pushing and withdrawing 1mL cold 1% FBS/PBS into the lungs *via* an angiocatheter (performed 3x) and then passed through a 70-micron filter (Falcon, Corning, NY). Cells were pelleted and resuspended in 2mL lysostaphin and then washed twice in 1% FBS/PBS. To determine total cell counts, the pellet was brought to 1mL before analysis. Cells were stained with PE anti-Ly6G (BioLegend) in 1% FBS/PBS for 30 minutes. Before analysis by flow cytometry, samples were washed 3x in 1%FBS/PBS. Samples were analyzed on a MACSQuant and data were analyzed with FlowJo software to determine fluorescent intensities, population percentages, and total counts.

### Colony forming units assays

BAL samples were stained with PE anti-Ly6G (BioLegend) and sorted into three populations: Ly6G^+^ATTO647N^+^GFP^+^, Ly6G^+^ATTO647N^+^GFP^-^, and Ly6G^+^ATTO647N^-^GFP^-^. Cell sorting was performed using a FACSAria (BD). *In vitro* assay samples omitted the PE anti-Ly6G stain and were sorted based on ATTO647N and GFP expression. Samples were pelleted and resuspended in 1mL of pH=11 H_2_O. Cells were lysed by incubating and vortexing for ~5 minutes (23). Lysates were serially diluted at 1:10 and plated, 3x10µL drops per dilution onto TSB agar plates. Plates were incubated at 37C overnight. Colonies were counted and the total CFU burden of the sorted cells was determined. CFU burden was normalized to the total events sorted in each population.

### Imaging

For imaging analysis of cell suspensions, cells were spun at 1500 RPM onto a superfrost plus glass slide (Fisher Scientific, Pittsburgh, PA) after phagocytosis and killing *in vitro*. *Ex vivo* analysis of lung tissue was performed through frozen sectioning of infected animals. WT animals were infected and euthanized twelve hours post-infection. The lungs were carefully removed avoiding puncture and inflated through the trachea with 0.8mL of a 2:1 mixture of optimal cutting temperature compound (OCT):PBS. The trachea was tied with a suture and the lungs were immediately frozen in liquid nitrogen. Once frozen, the tissue was then embedded in a mold with OCT (Sakura Finetek, Torrance, CA) on dry ice. Samples were sliced in a cryostat microtome at a thickness of 5-7 µm and adhered to superfrost plus glass slides. Prior to imaging, slides were fixed in cold acetone for 2 minutes and allowed to dry at room temperature.

For both types of imaging experiments, coverslips were applied with antifade mounting medium with DAPI (Vectashield, Newark, CA) and immediately imaged. Slides were imaged using a Nikon Eclipse 80i, with light provided by an X-Cite 120 lamp (Excelitas Technologies). Images were captured with a Retiga EXi Fast 1394 camera (QImaging, Burnaby, BC, Canada). Post-imaging analyses to merge images, analyze colocalization, and include scale bars were done using ImageJ.

In order to measure the colocalization of the GFP and ATTO647N fluorescence in the lung tissue, 11 images from distinct sections of lung tissue were analyzed (20x magnification). Images were collected by sectioning lungs from 3 separate animals. First, the background signal was subtracted from the GFP and ATTO647N images. The two images were compared with the “colocalization threshold” and “colocalization test” functions. Since we do not expect a relationship between the fluorescent intensity of the ATTO647N and GFP signal but do expect spatial correlation, we report Mander’s Coefficient for the GFP and ATTO647N channels relative to one another. The colocalization test was run with Costes approximation and 25 iterations of randomized pixels.

### Human neutrophil infections

Human cord blood was collected following approval of the Care New England Women & Infants Hospital IRB, Project number 04-0061. To isolate white blood cells, 4mL of cord blood was lysed with red blood cell lysis buffer (BioLegend) in the dark for 20 minutes. Cells were then washed 2x in assay buffer. Cells were suspended in 1.20mL of assay buffer and 100µLwas used per sample. Samples were inoculated with 15µL of bi-fluorescent *S. aureus* prepared as described above. Cells were incubated for 15 minutes at 37C, washed with warm lysostaphin, and brought to a final volume of 100uL. At the given time points (0, 30, 60, and 120 minutes) 10µL of the sample was plated into 200µL of cold PBS/FBS for analysis using a MACSQuant flow cytometer.

### Statistical analysis

Graphical depictions of the data represent group means and standard deviations (SD) generated using Prism 9 (GraphPad). The SD represents biological replicates across all figures except **Figure 3C**, in which the SD is representative of technical replicates calculating the CFU from one sample lysate for the distinct samples represented by each point on the plot. Where indicated, statistical analysis was conducted to compare groups. First, significance was calculated by the Prism 9 ordinary one-way ANOVA test. A p-value threshold of 0.05 indicated a significant difference when comparing the four groups (WT low, WT high, CGD low, CGD high) to one another using Tukey’s multiple comparisons tests. When there was no significant difference between the low and high dose for the same genotype but there was a significant difference between genotypes for each dose, this was represented graphically by grouping the genotypes under one line and indicating the asterisk for the highest multiple comparisons p-value determined by Tukey’s test. Asterisks indicate significance as follows: “ns” (not significant) for p>0.05, * for p<0.05, ** for p<0.01, *** p<0.001, and **** for p<0.0001.

## Results

### ATTO647N-NHS Ester fluorescently labels *S. aureus*

**Figure 1:**
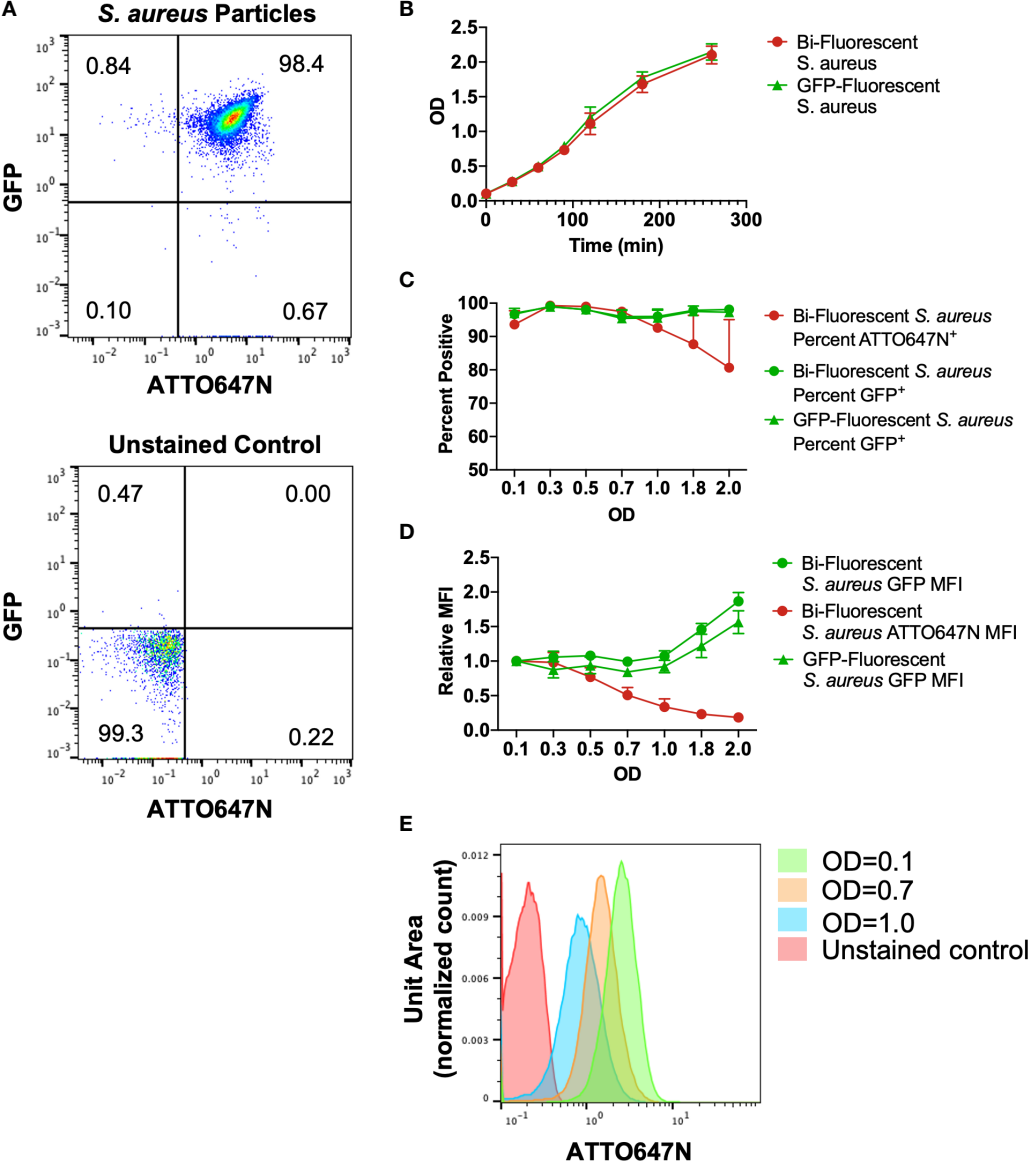
Characterization of cultured bi-fluorescent *S. aureus*. **(A)** Staining GFP-*S. aureus* with 200ng/mL ATTO647N-NHS Ester generates a pure population of bi-fluorescent *S. aureus* detectable by flow cytometry. **(B)** The growth curve of S. aureus with or without secondary ATTO647N-NHS Ester stain was indistinguishable. **(C)** The percent of particles gated as GFP^+^ or ATTO647N^+^ relative to unstained control demonstrates a loss of detection of the ATTO647N signal with bacterial replication. **(D)** Geometric mean fluorescent intensity (MFI) of GFP or ATTO647N signal relative to the signal at the start of growth in culture demonstrates robust GFP MFI and loss of ATTO647N signal with bacterial replication (n=3, performed across three independent experiments). **(E)** Representative shifts in the ATTO647N fluorescent intensity towards the unstained control with increasing OD, with overlap visualized at OD=1.0.

We found that staining superfolded GFP-producing *S. aureus* (18) with a low concentration of ATTO647N-NHS Ester generated a pure population of ATTO647N^+^GFP^+^ bi-fluorescent particles (**Figure 1A**). The stain did not affect *S. aureus* viability as the growth rate in culture was comparable to the unstained GFP-fluorescing *S. aureus* (**Figure 1B**). We predicted that with bacterial cell division, the ATTO647N mean fluorescent intensity (MFI) would decrease due to the dilution of the intracellular concentration of the dye. To determine the change in fluorescent intensity with proliferation, bi-fluorescent and GFP-fluorescent *S. aureus* cultures were grown from an initial optical density (OD) of 0.1. As expected, *S. aureus* stably expressing GFP maintained a detectable signal with culture growth, as particles remained nearly 100% GFP^+^ compared to a non-fluorescing, unstained *S. aureus* control (**Figure 1C**). The GFP MFI also remained robust with culture growth and was comparable between the bi-fluorescent and GFP-fluorescent groups (**Figure 1D**). The ATTO647N MFI began to decrease with growth in culture as the OD increased (**Figure 1D**). However, with the bacterial proliferation, the ATTO647N signal remained high enough to differentiate between stained and unstained *S. aureus* until an OD of 1.0 when the population begins to overlap with the unstained control (**Figure 1E**). Overall, the ATTO647N signal of conjugated *S. aureus* remains detectable after a substantial level of bacterial replication, without affecting *S. aureus* growth, making it an optimal candidate as a secondary marker for the assessment of bacterial killing by leukocytes.

### ATTO647N-NHS ester fluorescence is resistant to intraphagosomal bleaching

**Figure 2:**
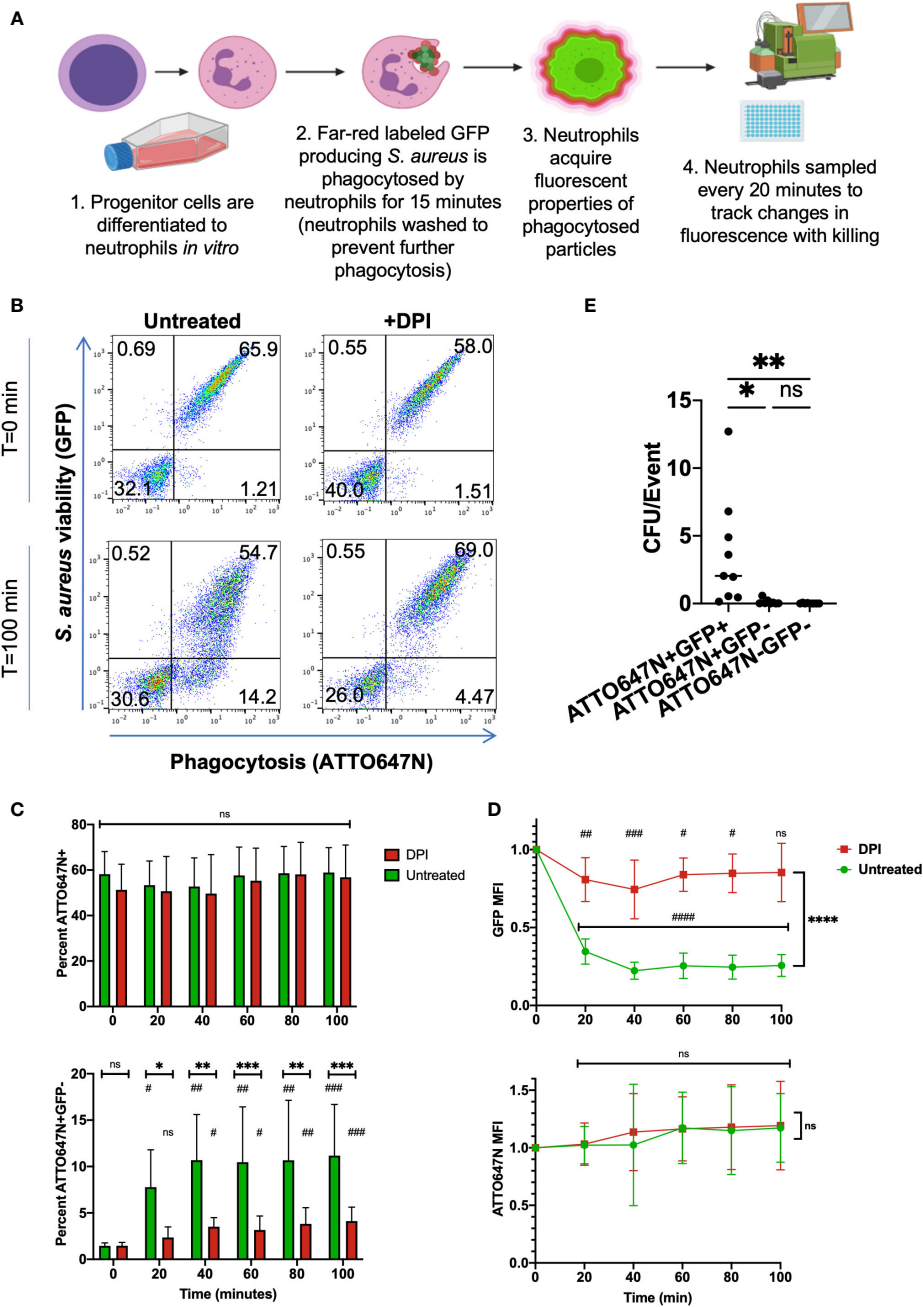
*In vitro* killing assay of bi-fluorescent *S. aureus.* **(A)** Schematic of *in vitro* screening process of bi-fluorescent *S. aureus.* **(B)** Immediately following phagocytosis, HB8 neutrophils are either ATTO647N^-^GFP^-^ or ATTO647N^+^GFP^+^. After 100 minutes, an ATTO647N^+^GFP^-^ population forms in the untreated group but not the DPI-treated group. **(C)** The percent of total ATTO647N^+^ HB8 neutrophils remains constant over time with no significant difference between untreated and DPI groups, or between the initial time point and subsequent time points within each group. Meanwhile, the percent of ATTO^+^GFP^-^ cells increases over time within each group (statistical significance indicated by #) and these changes are significantly greater in the untreated cells relative to the DPI treated cells at each time point (statistical significance indicated by *) **(D)** Quantification of GFP MFI within the ATTO647N^+^ events shows a dramatic loss of fluorescence over time in the untreated cells compared to the DPI treated cells and compared to the initial time point (the statistical significance between each time point relative to the initial time point is indicated by #; statistical significance between the untreated and DPI group is indicated by *). Meanwhile, the ATTO647N MFI remains consistent in both groups (n=9, performed across three independent experiments). **(E)** Bacterial burden from sorted cell lysates from both DPI treated and untreated cells, normalized to the total number of sorted events (n=9, performed across two independent experiments). “ns” (not significant) for p>0.05, * for p<0.05, ** for p<0.01, *** p<0.001, and **** for p<0.0001.

Conditional ectopic expression of transcription factor *Hoxb8* in murine bone marrow stem cells produces a progenitor cell line capable of exponential expansion while *Hoxb8* is induced or neutrophil differentiation through *Hoxb8* withdrawal (22). Differentiation of *Hoxb8* progenitors derived from a wild-type (WT) C57BL/6 mouse into mature neutrophils (HB8 neutrophils) was confirmed through characterization of surface markers. Differentiation of progenitors into neutrophils was confirmed by increased Ly6G expression and loss of cKit expression by HB8 neutrophils (**Supplemental Figures 1A, B**). HB8 neutrophils produce ROS and have microbial killing capacity *in vitro* (20), making them a useful screening tool for the effects of phagocytosis and intracellular killing of the bi-fluorescent *S. aureus*. For preliminary experiments, the bi-fluorescent *S. aureus* was phagocytosed by HB8 neutrophils *in vitro*, treated with lysostaphin to remove residual extracellular *S. aureus* (24), and changes in fluorescence were monitored over time as intracellular killing occurred (**Figure 2A**). Treating cells with cytochalasin D before inoculation to prevent phagocytosis by disrupting the actin cytoskeleton blocked neutrophils from acquiring the GFP and the ATTO647N signal, confirming that the detected fluorescence is predominantly acquired by internalizing *S. aureus* (**Supplemental Figures 2A, B**).

At the initial time point following phagocytosis, the HB8 neutrophils with engulfed *S. aureus* acquired the bi-fluorescent properties, as measured by flow cytometry (**Figure 2B**). The percentage of ATTO647N^+^ events was stable over time despite changes in the percentage of GFP^+^ events (**Figure 2C**). As expected, the percentage of GFP^+^ events and GFP MFI decreased over time in HB8 neutrophils, suggesting successful intracellular *S. aureus* killing (**Figure 2D**). Importantly, these metrics remained the same over time in the ATTO647N channel (**Figure 2D**). HB8 neutrophils treated with diphenyleneiodonium (DPI), to inhibit the generation of ROS, exhibit a robust GFP signal that is maintained over time (**Figures 2B–D**). The bleaching of the GFP signal, but not the ATTO647N signal, suggests that the latter fluorophore is resistant to quenching by neutrophils and remains stable in the conditions of the active phagosome.

Taken together, these results suggest that ATTO647N fluorescence identifies the population of cells that have undergone phagocytosis of *S. aureus* and GFP fluorescence indicates which of those cells contain viable *S. aureus*. Therefore, we can group cells that have encountered the bi-fluorescent *S. aureus* into three categories: ATTO647N^+^GFP^+^ cells which have viable intracellular *S. aureus*, ATTO647N^+^GFP^-^ cells which have non-viable intracellular *S. aureus*, and ATTO647N^-^GFP^-^ cells which have not phagocytosed *S. aureus*.

### Fluorescent properties of neutrophils reflect intracellular *S. aureus* viability

To validate that the ATTO647N and GFP fluorescence associated with neutrophils represent the expected intracellular *S. aureus* viability, we exposed neutrophils to bi-fluorescent *S. aureus* and then sorted cells within the three quadrants described above. After allowing 120 minutes for intracellular killing of ingested *S. aureus*, both untreated and DPI-treated HB8 neutrophils were sorted, lysed, and plated on solid media to quantify the number of viable *S. aureus*. Cells were lysed using deionized water with a pH of 11 to release all viable intracellular bacteria and ensure that *S. aureus* colonies were not under-represented due to impaired dispersal on the agar plate (23). The number of *S. aureus* colony forming units (CFUs) was normalized to the number of events (HB8 neutrophils) that were sorted from the bulk population. Both the ATTO647N^+^GFP^-^ and ATTO647N^-^GFP^-^ sorted cell populations yielded negligible CFU (**Figure 2E**). Meanwhile, the sorted ATTO647N^+^GFP^+^ cells contained significantly more CFUs confirming the presence of viable intracellular *S. aureus* (**Figure 2E**). These data also validate that the quenching of GFP is a reliable marker for neutrophils that have completed intracellular killing of *S. aureus*.

**Figure 3:**
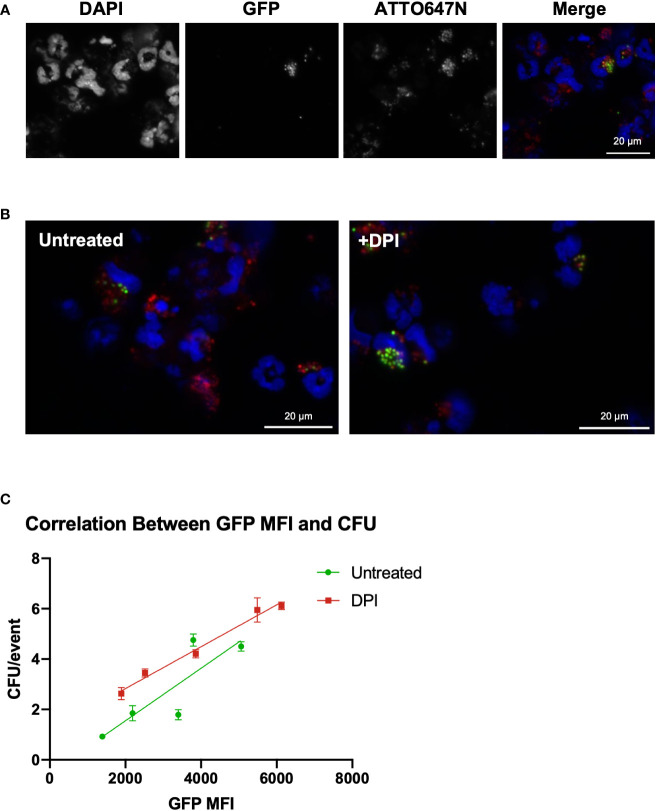
GFP fluorescent intensity correlates with the number of viable internalized *S. aureus* cocci. **(A)** Imaging of HB8 neutrophils after 2 hours of killing *in vitro* (40x). **(B)** GFP/ATTO647N merged images for untreated and DPI treated HB8 neutrophils (40x). **(C)** Correlation between GFP MFI and viable intracellular bacterial burden (CFU representative of plating lysate in triplicate).

Next, we investigated whether the GFP MFI was related to intracellular *S. aureus* viability. Microscopic imaging of HB8 neutrophils after 120 minutes of intracellular killing in suspension provides insight into the variability of neutrophil phagocytosis and killing (**Figures 3A, B**). Within the bulk population, there are HB8 neutrophils that have phagocytosed different quantities of *S. aureus* particles, as indicated by the broad range of ATTO647N fluorescent intensity by flow cytometry (**Figure 2B**) and visualized microscopically (**Figures 3A, B**). Accordingly, in the untreated HB8 neutrophil population some cells have both viable (GFP^+^) and non-viable (GFP^-^) particles internalized and potentially within the same phagolysosome (**Figure 3B**). Meanwhile, based on GFP fluorescence, nearly all *S. aureus* is near completely viable DPI-treated cells (**Figure 3B**). These images, in conjunction with the findings of Schwartz, et al., establish a positive relationship between GFP fluorescence and *S. aureus* cocci within neutrophils suggesting that GFP MFI may provide insight into the intracellular *S. aureus* burden of individual cells or bulk populations. We expect that cells or populations with a higher MFI have a greater bacterial burden despite some degree of variability in the fluorescent intensity of the original *S. aureus* population (**Figure 1A**).

To generate cell populations with various MFIs, we inoculated HB8 neutrophils with an increasing multiplicity of infection and sorted the ATTO647N^+^GFP^+^ population of neutrophils 120 minutes after allowing the intracellular killing to occur. As expected, we found that the cell populations with higher GFP MFI had a greater intracellular bacterial burden (**Figure 3C**). We also wanted to determine whether the GFP MFI of DPI-treated neutrophils was proportionately representing viable intracellular bacterial burden, a key question given that GFP quenching is ROS-dependent (17). We found the correlation between GFP MFI and CFUs from the DPI-treated sample was comparable to that of the control, supporting the notion that GFP MFI is a robust parameter for quantifying intracellular bacterial burden (**Figure 3C**).

### Application of bi-fluorescent *S. aureus* to demonstrate neutrophil dysfunction

Chronic granulomatous disease (CGD) is marked by neutrophil dysfunction due to a loss of function of the NADPH oxidase responsible for superoxide production in the phagolysosome. Since *S. aureus* killing is dependent on NADPH oxidase activity, hosts with CGD are disproportionately susceptible to *S. aureus* infections (7). Loss-of-function mutations in the x-linked *CYBB* gene encoding the gp91^phox^ subunit are the most common cause of CGD. The widely used murine CGD model harbors knockout of the *Cybb* gene leading to loss of ROS production by neutrophils. CGD mice recapitulate the human disease well, including the impaired clearance of *S. aureus* infections (25). Therefore, we used this disease model to assess the *in vivo* use of the bi-fluorescent *S. aureus* to quantify neutrophil killing capacity.

**Figure 4:**
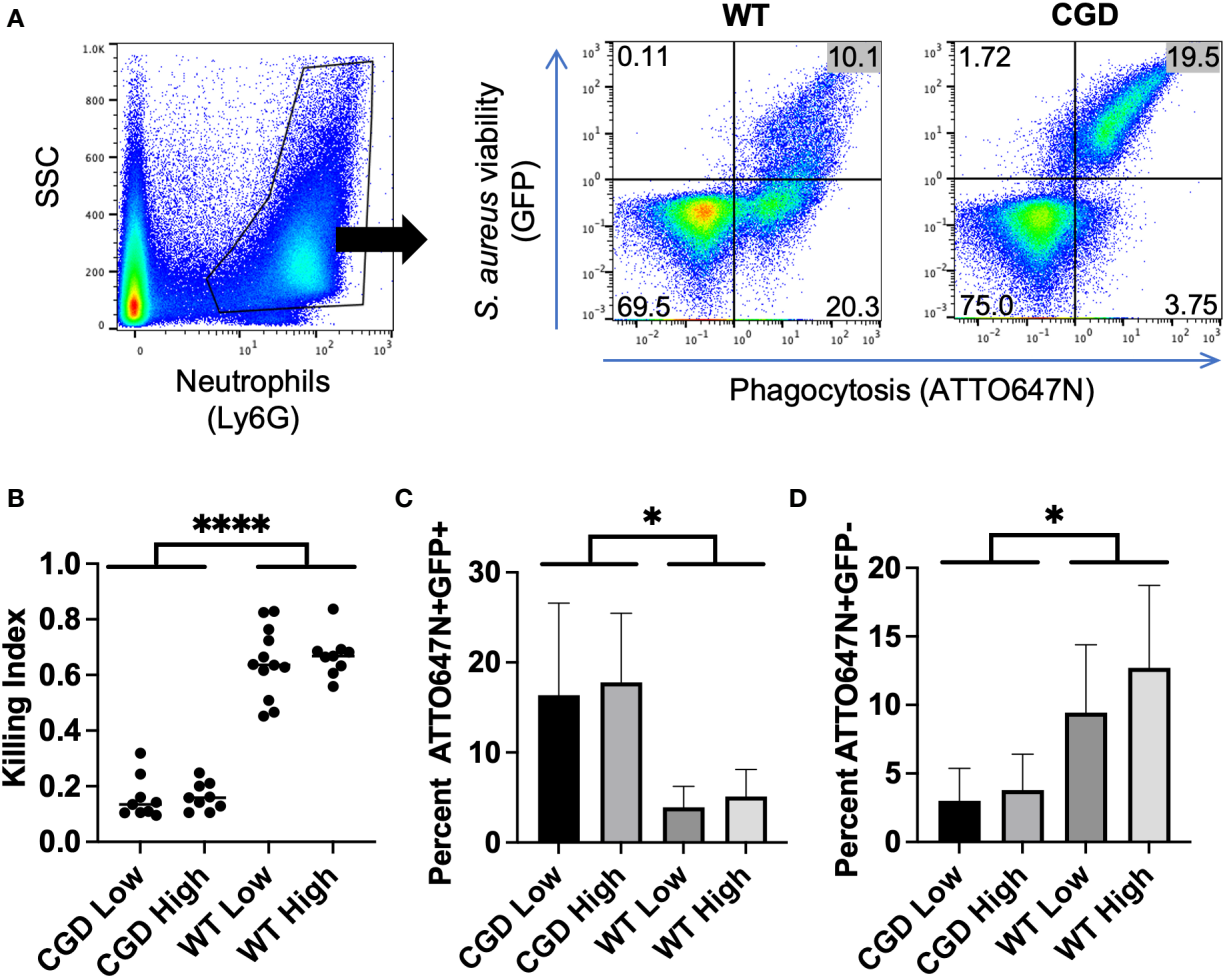
BAL from WT and CGD animals infected with bi-fluorescent *S. aureus.* **(A)** Gating on Ly6G^+^ events within the BAL enables the separation of neutrophils based on GFP and ATTO647N fluorescence. **(B)** The ratio of ATTO647N^+^GFP^-^ cells to all ATTO647N^+^ cells represents the neutrophils that have completely killed the *S. aureus* compared to those which have undergone phagocytosis: the killing index. **(C)** Percent of neutrophils that are ATTO647N^+^GFP^+^ indicated the intracellular burden of viable *S. aureus*. **(D)** Percent of neutrophils that are ATTO647N^+^GFP^-^ indicates the population of cells that have completed killing (n=8-11 animals per group, from 7 independent experiments). * for p<0.05 and **** for p<0.0001.

We harvested samples from wild-type (WT) and CGD mice twelve hours after pulmonary infection with a low (6x10^7^ CFU) or high (8x10^7^ CFU) inoculum of bi-fluorescent *S. aureus*. Since the ATTO647N signal dilutes with significant bacterial replication, we recognize that bi-fluorescent *S. aureus* is primarily applicable to analyzing the acute response to infection. Bronchoalveolar lavages (BAL) were collected and stained with anti-Ly6G to identify the neutrophils *via* flow cytometry (**Figure 4A**). Very few Ly6G^-^ events were GFP^+^ or ATTO647N^+^ indicating that neutrophils were the predominant cell type responsible for phagocytosis of *S. aureus* in the airspaces at this time point (**Supplemental Figure 3**).

After gating for Ly6G^+^ events to identify neutrophils within the BAL, we were able to assess their *S. aureus* killing capacity based on the GFP and ATTO647N fluorescence associated with neutrophils (**Figure 4A**). Since the percent of neutrophils that engage in phagocytosis varies between experiments, we calculated a killing index defined as the percent of neutrophils that had completed killing relative to the percentage of neutrophils engaged in phagocytosis (ATTO^+^GFP^-^/ATTO^+^GFP^+/-^). Based on this metric, populations with a ratio closer to 1 represent greater success in killing *S. aureus* relative to populations closer to 0. We observed that the killing index of CGD neutrophils was significantly lower than that of the WT controls (**Figure 4B**).

**Figure 5:**
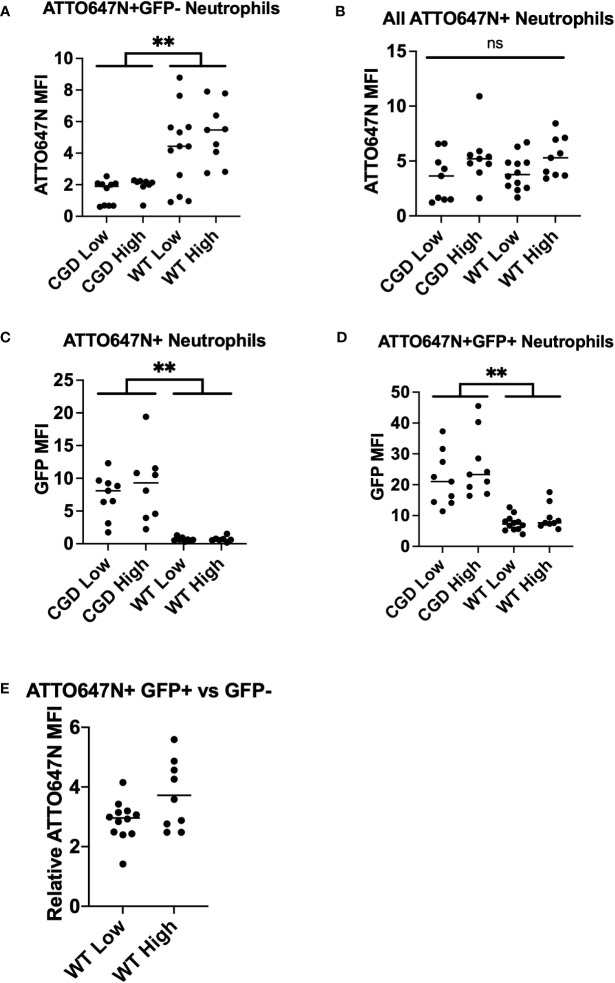
Analysis of BAL neutrophil subpopulation fluorescent intensities. **(A)** WT neutrophils have increased ATTO647N MFI for neutrophils that have completed killing, indicating clearance of larger bacterial burdens on a per-cell basis. **(B)** The neutrophil ATTO647N MFI was comparable between groups implying similar levels of phagocytosis. **(C)** The GFP MFI for all ATTO647N^+^ events demonstrate an increased bacterial burden in phagocytosing CGD neutrophils relative to WT neutrophils. **(D)** GFP MFI of the ATTO647M^+^GFP^+^ population demonstrates the same trend of increased intensity in CGD animals. **(E)** The relative ATTO647N MFI was calculated by dividing the ATTO647N MFI of ATTO647N^+^GFP^+^ events by ATTO647N^+^GFP^-^ events (n=8-11 animals per group, from 7 independent experiments). “ns” (not significant) for p>0.05, ** for p<0.01.

The fraction of BAL neutrophils within each ATTO647N/GFP quadrant also behaved as expected based on genotype. The percentage of neutrophils containing viable *S. aureus* (ATTO647N^+^GFP^+^) was greater for the CGD animals than for the WT animals (**Figure 4C**). Since CGD leads to increased recruitment of neutrophils to the BAL (6, 25), we also compared the total number of neutrophils within each gate. Again, the CGD host trended towards a higher total neutrophil count compared to WT mice (**Supplemental Figure 4**). This demonstrates a larger intracellular burden of viable *S. aureus* in CGD BAL neutrophils, accompanied by a diminished population of ATTO647N^+^GFP^-^ neutrophils that had completed killing *S. aureus* (**Figure 4D**). We speculate that the small population of ATTO647N^+^GFP^-^ cells in the CGD animals may represent the phagocytosis of dead *S. aureus* or the successful employment of non-oxidative mechanisms to kill *S. aureus*. *In vitro*, we observed that both WT and CGD neutrophils readily ingest heat-killed *S. aureus* and acquire ATTO647N fluorescence (**Supplemental Figure 5**). It is also possible that non-oxidative killing mechanisms are effective in cells with a low level of *S. aureus* phagocytosis, as the ATTO647N^+^GFP^-^ neutrophil population in CGD mice has a significantly lower ATTO647N mean fluorescent intensity compared to the same population from WT mice (**Figure 5A**).

Having shown that the GFP MFI of neutrophils that have taken up *S. aureus* corresponds to the viable intracellular burden of *S. aureus*, we performed further analyses of BAL neutrophils from WT and CGD mice that were infected with bi-fluorescent *S. aureus*. We observed a similar relationship *in vivo* as was established *in vitro*, suggesting that BAL neutrophils with a greater GFP MFI contain a greater *S. aureus* bacterial burden (**Figure 3C**; **Supplemental Figure 6**). Comparing the ATTO647N MFI for all ATTO647N^+^ events, we observed no differences across groups (**Figure 5B**), implying that the degree of phagocytosis is comparable between the WT and CGD neutrophils. We first looked at the GFP MFI of all neutrophils involved in phagocytosis to understand the overall population activity. The CGD ATTO647N^+^ neutrophils had a significantly higher GFP MFI compared to the analogous population of neutrophils in WT animals, further supporting the conclusion that the population of neutrophils with viable intracellular *S. aureus* is greater in the CGD animals (**Figure 5C**). Next, we analyzed the GFP MFI of only the cells with viable intracellular *S. aureus* (ATTO647N^+^GFP^+^) and again observed a greater GFP MFI for CGD neutrophils relative to WT (**Figure 5D**). We recognize that the analysis of ATTO647N^+^GFP^+^ may be biased if the GFP MFI of cells with a lower intracellular burden are more efficient at killing (and transitioning to the GFP^-^ population), which may lead to a higher GFP signal in the remaining population of cells. However, MFI information from ATTO647N^+^GFP^+^ neutrophils is valuable to understanding the state of cells which are unable to complete ROS-dependent intracellular killing of *S. aureus*. In BAL neutrophils from WT mice, we also observe that the ATTO647N MFI is higher in the ATTO647N^+^GFP^+^ population than in the ATTO647N^+^GFP^-^ population (**Figure 5E**), supporting the observations of others that neutrophils with increased intracellular *S. aureus* burden have decreased intracellular killing (17).

### Bi-fluorescent *S. aureus* allows localization within the airspace *in vivo*

**Figure 6:**
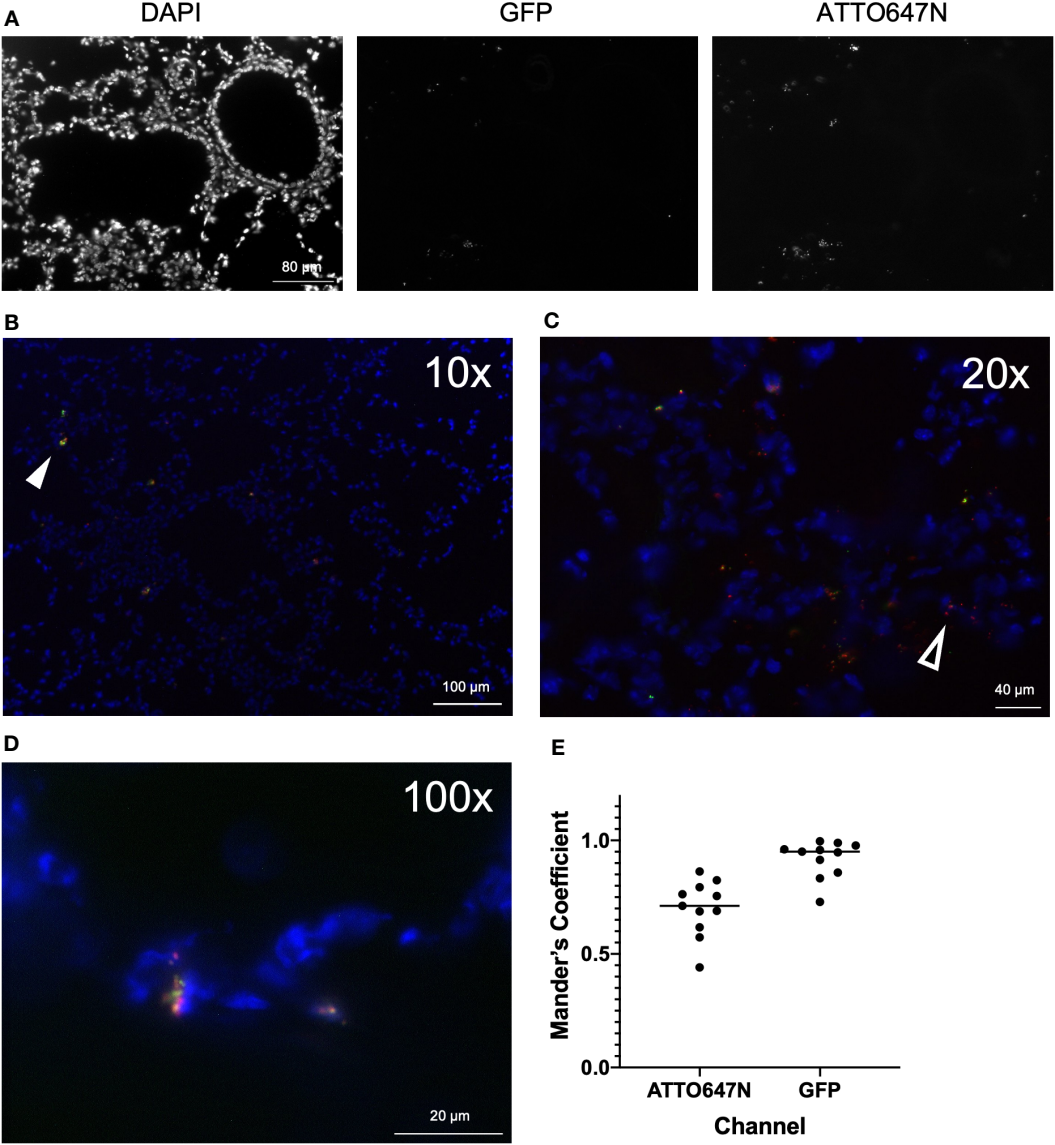
Imaging of airways post-infection with bi-fluorescent *S. aureus*. **(A)** Frozen lung sections were prepared from WT mice (n=3) 12 hours post-infection with bi-fluorescent *S. aureus* to visualize the infection in the tissue. Unmerged DAPI, GFP, and ATTO647N images show localization of airway cells, viable *S. aureus*, and total *S aureus*, respectively (20x). Merged images at **(B)** 10x **(C)** 20x and **(D)** 100x visualize both killed (red, open arrowhead) and viable (yellow, arrowhead) *S. aureus* within the tissue. **(E)** Colocalization analysis of GFP and ATTO647N images (n=11) show high overlap of the GFP signal with the ATTO647N signal (Mander’s Coefficient ~1) and incomplete overlap of the ATTO647N signal with the GFP signal (Mander’s Coefficient <1).

Analyses to probe the localization of cells laden with *S. aureus* within the context of the surrounding tissue environment provide insight into the dynamics of the host response. To visualize bi-fluorescent *S. aureus in vivo*, we imaged frozen sections from WT mice twelve hours post-infection. Merging DAPI, ATTO647N, and GFP images enabled the identification and localization of viable and nonviable *S. aureus* (**Figures 6A–D**). We again often observed viable cocci with larger clumps or clusters compared to the non-viable ones (**Figure 6D**).

Visualization of infected lung tissue by microscopy emphasizes the presence of cocci throughout, as opposed to analysis of killing on a single-cell level by flow cytometry. Images were analyzed to determine the degree of colocalization of the GFP and ATTO647N fluorescent signal. Mander’s coefficient was calculated to quantify spatial overlap for GFP and ATTO647N. We expected that Mander’s coefficient for the GFP channel would be equivalent to one, since anywhere GFP-expressing viable *S. aureus* is detected, the ATTO647N fluorescent signal should also be present. As anticipated, the average Mander’s coefficient for the GFP channel was 0.92 +/- 0.08 (**Figure 6E**). The Mander’s coefficient in the ATTO647N channel ranged from 0.44 to 0.86 (**Figure 6E**). This value roughly represents the proportion of cocci that are still viable, marked by the overlapping GFP signal. This proportion is higher compared to the proportion of cells with viable internalized *S. aureus* measured by the killing index or GFP fluorescence intensity measured by flow cytometry (**Figures 4**, **5**). However, the Mander’s coefficient is representative of all cocci, both intracellular and extracellularly located, while the flow cytometry analysis only measures intracellular killing. This difference also highlights the differences in looking at *S. aureus* killing on a per-cell basis versus a per-cocci basis and further supports the notion that cells heavily loaded with cocci may have impaired killing. Finally, differences in the killing index and Mander’s coefficient may represent differences in the killing capacity of cells found in the BAL as opposed to whole tissue.

### Bi-fluorescent *S. aureus* is killed by human leukocytes

Animal models of infection have obvious benefits for studying disease *in vivo*. However, findings in the murine host do not always translate to human pathophysiology. To investigate whether the assay we have extensively characterized using murine neutrophils translates to human cells, we conducted a screen of the bi-fluorescent *S. aureus* in human leukocytes. Cells were obtained from freshly drawn umbilical cord blood samples that were subjected to red blood cell lysis and then inoculated with bi-fluorescent *S. aureus* to characterize changes in fluorescence over time.

**Figure 7:**
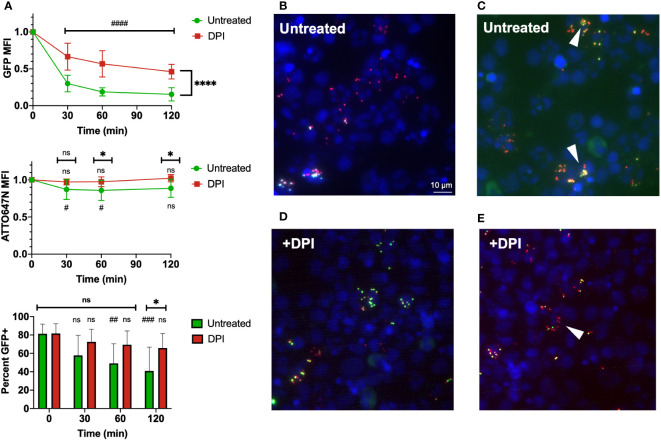
Use of bi-fluorescent *S. aureus* to model infection in human neutrophils. **(A)** Human leukocytes inoculated with bi-fluorescent *S. aureus* maintain the ATTO647N fluorescent signal while quenching the GFP fluorescent intensity over time based on intensity and percent GFP^+^ cells, statistical differences between groups indicated with a * and differences within each group relative to the initial time point indicated with a # (n=8, performed across three independent experiments). **(B)** Imaging (60x) performed two hours after human cells were infected *in vitro* demonstrates cells with few cocci broadly kill the internalized *S. aureus* and fluoresce as ATTO647N^+^ (red). **(C)** Cells that have viable *S. aureus* internalized ATTO647N^+^GFP^+^ (yellow, arrowheads) often have internalized larger clumps of *S. aureus*. **(D)** DPI treated human cells largely have viable (yellow) internalized *S. aureus* regardless of cocci internalized. **(E)** Some DPI treated cells still quench the GFP fluorescent signal (red, arrowhead). “ns” (not significant) for p>0.05, * for p<0.05 and **** for p<0.0001.

The use of the ATTO647N-Ester conjugation of *S. aureus* to identify cells that have undergone *S. aureus* phagocytosis translates well to human cells, as we observed that leukocytes maintained a robust ATTO647N MFI over time (**Figure 7A**). Again, the GFP fluorescence intensity decreased over time, suggesting intracellular killing of *S. aureus* within the untreated group. These findings were confirmed *via* microscopic imaging of cells two hours after allowing phagocytosis and intracellular killing to occur in suspension. Many cells contain particles with ATTO647N fluorescence (red) in the absence of GFP (**Figure 7B**). The areas which did have viable *S. aureus*, determined by co-localized ATTO647N and GFP (yellow), were predominantly present in cells that had likely had multiple phagolysosomes based on their distribution. The DPI-treated samples largely showed viable *S. aureus* within cells (**Figure 7D**). Interestingly, the GFP MFI decreased more than expected—though not to the degree of the untreated cells—in the DPI-treated cells (**Figures 7A, E**). This apparent decrease in GFP fluorescence intensity was also accompanied by a slight decrease in percent GFP^+^ events within the ATTO647N^+^ population (**Figure 7C**). Taken together, these data indicate that bi-fluorescent S. aureus reports its internalization and eradication by human leukocytes.

## Discussion

The rapid and voluminous response of neutrophils to *S. aureus* infection is critical to halt its progression from its initial site. During the acute stages of the innate immune response, there is a complex orchestration of cellular host defense as the inflammatory environment develops. Early events can quickly shift the microbial burden towards containment and eradication, or, in cases of inadequate immunity, towards the expansion and dissemination of infection. Understanding the innate immune response to *S. aureus* as it occurs at tissue sites requires innovative techniques for measuring cell function against microbes. In this study, we have further developed a method to detect and quantify the intracellular eradication of *S. aureus* and demonstrate its utility *in vivo* and at the resolution of individual cells.

The use of bi-fluorescent *S. aureus* to quantify its intracellular killing *in vivo* provides convenient insight into the mechanism and dynamics of neutrophil phagocytosis and microbicidal activity of the phagosome. Single-cell analysis of killing activity provides a valuable tool for better understanding the role of cellular subpopulations in bacterial clearance *in vivo*. The relevant applications of this adapted technique include neutrophil heterogeneity studies in pathological and non-pathological states (26–31). Despite a growing understanding of the existence of different subpopulations of neutrophils, much remains unknown as to the relation between characterized differences and influence on function. Tsuda et al. previously identified three subsets of neutrophils in the context of susceptibility to *S. aureus* infection. Despite variable surface markers and cytokine production profiles, subsets were described to have similar *in vitro* MRSA killing capabilities (29). However, when subject to the *in vivo* environment these neutrophil subsets may respond differently to the cytokines they produce or their ability to crosstalk with other immune cells, modulating their bactericidal activity. Characterization of the neutrophil subpopulation which readily engulfs and kills *S. aureus*—as identified by the ATTO^+^GFP^-^ population in the assay we characterize here—may provide insight for developing therapies that support or augment innate immune function.

In the study that first characterized GFP-expressing *S. aureus* as a tool for quantifying its intracellular viability, Schwartz et al. described amplified resistance of *S. aureus* to eradication as the number of cocci within the neutrophil increased *in vitro* (17). While validating the use of this assay, we observed a similar trend *in vivo* as neutrophils with viable intracellular *S. aureus* were predominantly those that had engulfed a greater number of *S. aureus.* The relatively large population of neutrophils that have not undergone phagocytosis but are present in the BAL provokes questions as to why some cells engulf more *S. aureus* than they can efficiently kill while others do not internalize any. In studies of competitive phagocytosis, others have described phagocytosis as a “nonrandom” event, with a subset of neutrophils more likely to undergo phagocytosis (32). There is even apparent variability within a single neutrophil, as the production of HOCl within the multiple phagolysosomes of the same cell can occur with widely disparate lag time after formation (26). *S. aureus* has also evolved to evade or neutralize host defenses. For example, the production of superoxide dismutase by *S. aureus* converts the free radicals in the phagolysosome to water and oxygen (15). Therefore, targeting neutrophils to improve their killing capacity through modulating their degree of phagocytosis or intracellular ROS production may improve clearance of infection.

We anticipate that in extending the capability of a *S. aureus* killing assay with single-cell resolution will also enable further pursuit of questions that remain in understanding the mechanisms by which neutrophils eradicate microbes intracellularly. There has been debate as to the precise role of the biochemical pathways and products of the NADPH oxidase in the phagosome. Specifically, there are questions about whether ROS are responsible for direct toxicity towards microbes or confer their antimicrobial capacity predominantly by buffering the phagolysosome for optimal non-oxidative killing activity (e.g., by serine proteases) (10, 12). Our results correlating intracellular CFU and GFP MFI are consistent with the idea that HOCl production, as reported by GFP bleaching, is tightly correlated with the killing of internalized *S. aureus*. However, further investigation to delineate the necessity of proteolytic enzymes is warranted and having a means to quantify both *S. aureus* burden and viability will provide additional insight to solve questions that remain.

Within this study, we included a low and high dose of *S. aureus*, distinguished as being non-lethal and lethal doses, respectively, in CGD mice. The activity of the neutrophils that are recruited to the infected airspaces of the lung was independent of dose, as there were no statistical differences in the fraction of neutrophils that had completed *S. aureus* killing or the neutrophil GFP MFI between mice of the same genotype receiving the different initial bolus of *S. aureus*. This indicates that the activity of neutrophils was dependent on genotype, but not necessarily on potential differences in the environment during the acute response to low versus the high burden of infection. As pathologic resistance to conventional antimicrobial agents steadily increases, methods such as this bi-fluorescent killing assay to study the *in vivo* dynamics of cell-mediated antimicrobial activity will be of the utmost importance to enhance the pursuit of innovative therapeutic development.

Relatedly, it is important to understand how the use of biological or pharmaceutical interventions to treat infection occurs in conjunction with the host’s immune response. For example, antibiotics that interfere with the effector functions of immune cells may in turn have detrimental effects despite potent antimicrobial activity *in vitro* (33, 34). Conversely, treatments can have a synergistic effect or support the immune system’s intrinsic response leading to better outcomes than expected based on *in vitro* results (35–37). Therefore, when testing and developing treatments, *in vivo* models of infection should be prioritized. This is particularly relevant for therapies targeting *in vivo* evasion tactics of *S. aureus*, which employs numerous mechanisms to downregulate inflammation, avoid internalization, neutralize anti-microbial factors, and induce neutrophil cytolysis (15, 38). Multiple factors produced by *S. aureus* within the phagolysosome neutralize the antimicrobial components generated by the neutrophil. Anti-virulence therapies are gaining traction but also need to be understood in the context of the host response (39, 40). Consequently, methods to understand and quantify changes in immune-mediated microbial clearance are vital for developing and testing these much-needed new approaches to antimicrobial therapy.

## Data availability statement

The raw data supporting the conclusions of this article will be made available by the authors, without undue reservation.

## Ethics statement

The studies involving human participants were reviewed and approved by Institutional Review Board, Care New England Women & Infants Hospital. Written informed consent for participation was not required for this study in accordance with the national legislation and the institutional requirements. The animal study was reviewed and approved by Lifespan Animal Welfare Committee, Rhode Island Hospital.

## Author contributions

KH: Designing research studies, conducting experiments, analyzing data, writing the manuscript. SL-N: Cord blood collection. JC: Designing research studies, conducting experiments, analyzing data. MM: Designing research studies, conducting experiments, analyzing data. JB: Supervising the project, designing research studies. AH: Developing GFP-*Staphylococcus aureus* strain. CL: Supervising the project, designing research studies, conducting experiments, analyzing data, writing the manuscript. All authors contributed to the article and approved the submitted version.
