# Supplementary material for: Bi-fluorescent Staphylococcus aureus infection enables single-cell analysis of intracellular killing in vivo
Source: Front Immunol. 2023 Jan 23;14:1089111. doi: 10.3389/fimmu.2023.1089111 (PMC9900177; doi:10.3389/fimmu.2023.1089111)
Supplement: Supplementary file 1 [file DataSheet_1.pdf]

Bi-Fluorescent *Staphylococcus aureus* infection enables single-cell analysis of intracellular killing *in vivo*

Kristina D. Hinman, Sonia S. Laforce-Nesbitt, Joshua T. Cohen, Miles Mundy, Joseph M. Bliss, Alexander R. Horswill, Craig T. Lefort

Supplementary Information

Supplemental Figure 1

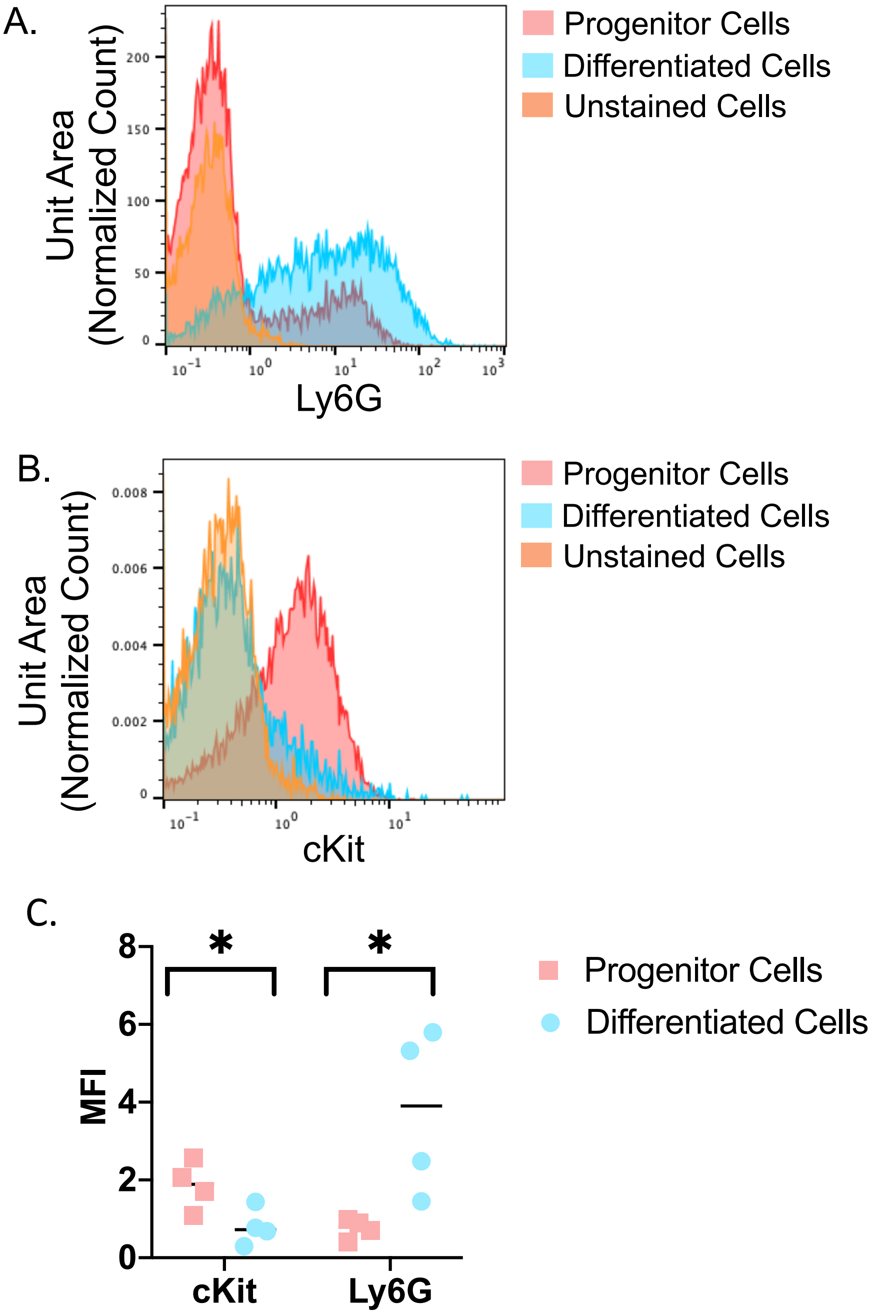

Supplemental Figure 1: Differentiation of HoxB8 derived neutrophils was monitored by (A) increased expression of Ly6G and (B) loss of cKit expression. (C) Quantification of differentiation can be monitored by changes in Ly6G and cKit by MFI (n=4, four independent experiments).

Supplemental Figure 2

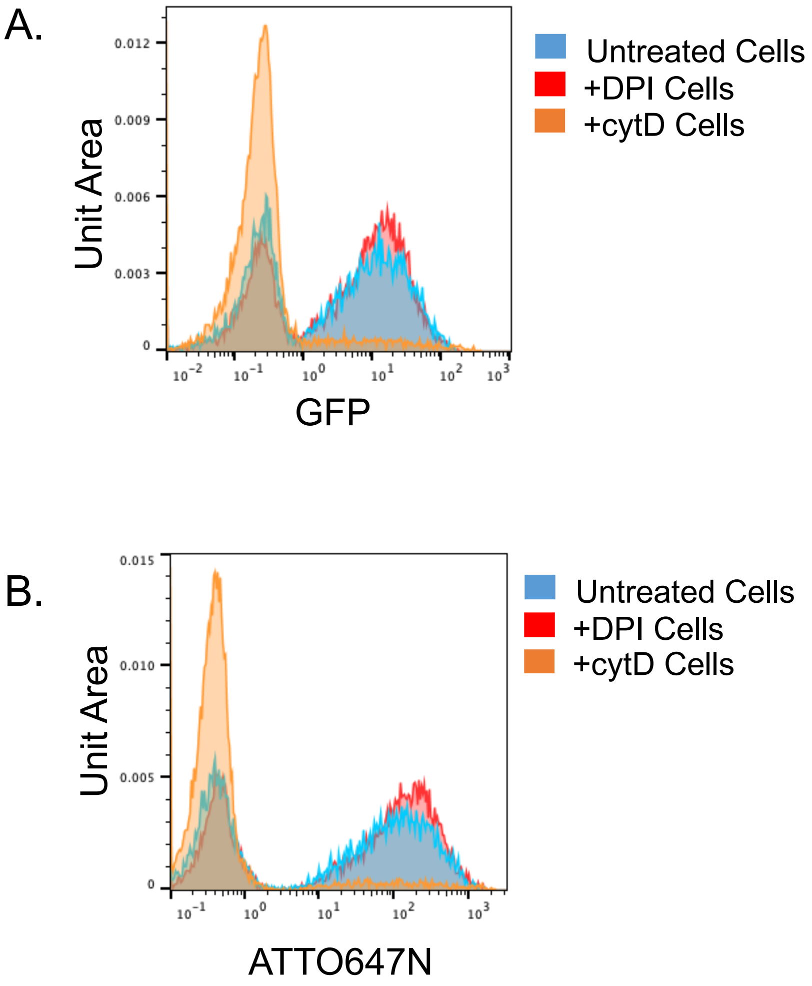

Supplemental Figure 2: Gain of fluorescence represents internalized *S. aureus*. Samples were incubated with bi-fluorescent *S. aureus* for 60 minutes and then prepared for analysis as described in the Methods. Cells pretreated with cytochalasin D had negligible fluorescence in either the (A) GFP channel or (B) ATTO647N channel.

Supplemental Figure 3

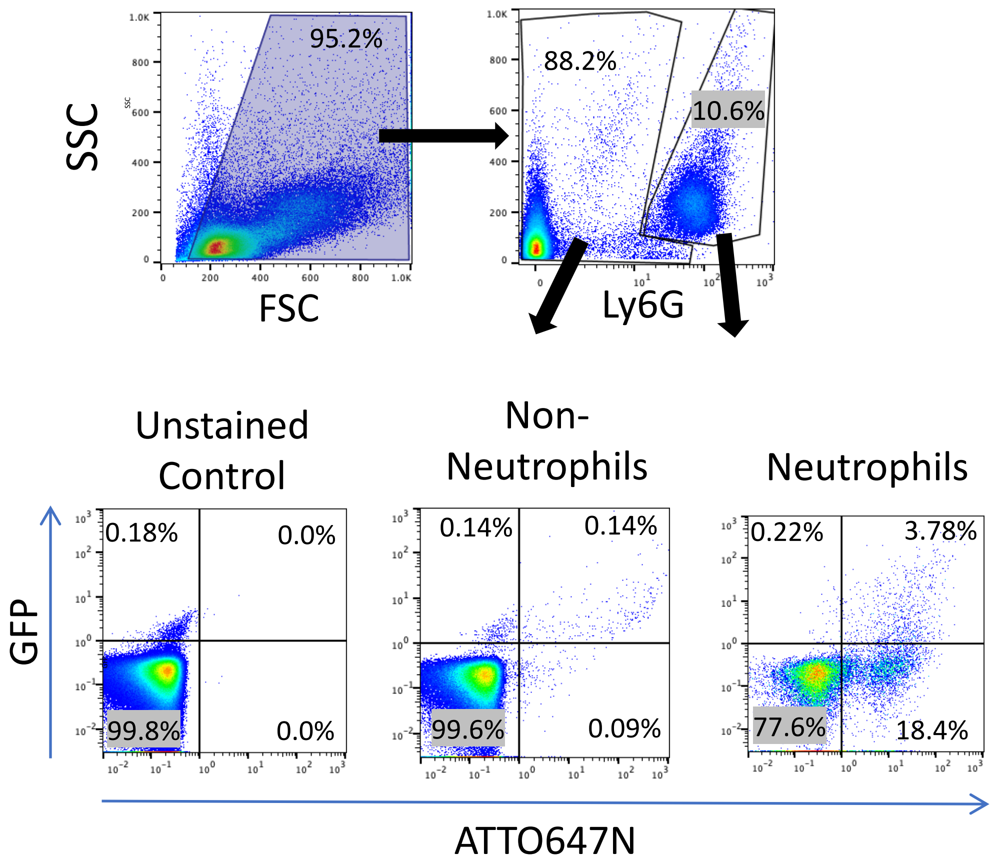

Supplemental Figure 3: Neutrophils represent the majority of cells with internalized *S. aureus* 12 hours post-infection. BAL samples were collected from infected mice and analyzed by flow cytometry. Gating on Ly6G+ and Ly6G- populations and comparing the fraction of ATTO647N+ cells shows that a very small percent of BAL non-neutrophils (Ly6G-) internalize *S. aureus*.

Supplemental Figure 4

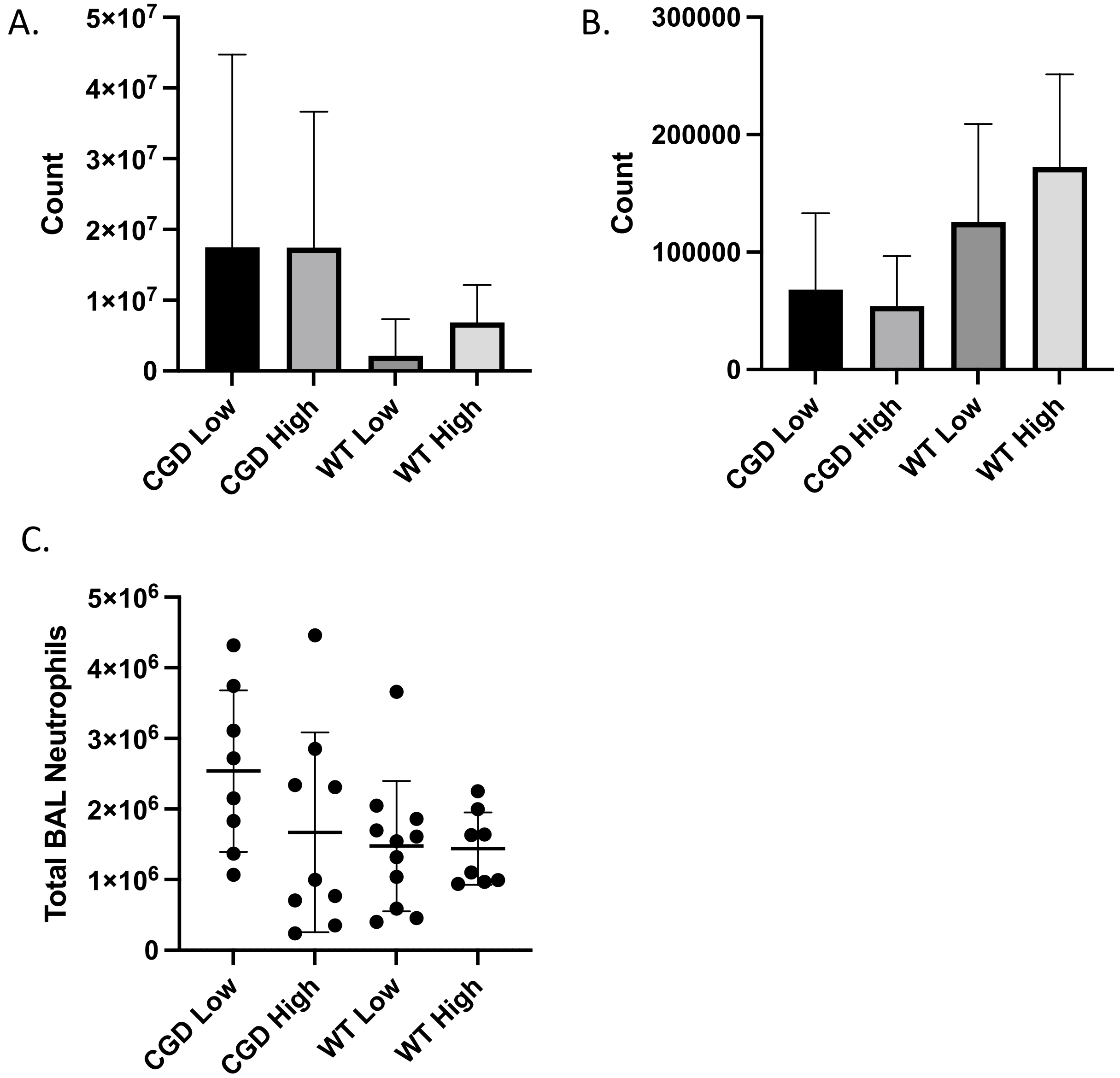

Supplemental Figure 4: Total neutrophil counts within each ATTO647N/GFP gate. (A) Neutrophil ATTO647N+GFP+ BAL counts represent live intracellular *S. aureus* total burden. (B) ATTO647N+GFP- neutrophil events represent killed intracellular *S. aureus* total count. (C) Total BAL neutrophil count trends towards increased counts in the CGD animals.

Supplemental Figure 5

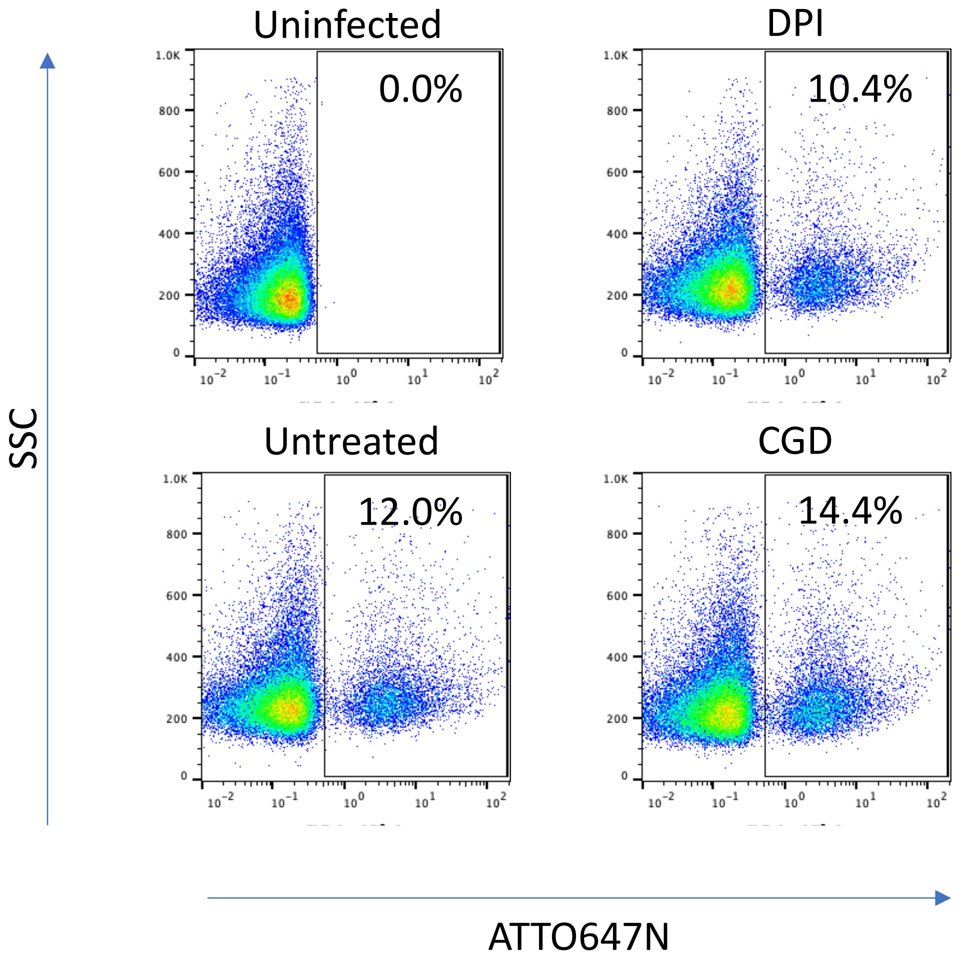

Supplemental Figure 5: Neutrophils phagocytose dead *S. aureus in vitro*. HB8 cells were incubated with heat-killed *S. aureus*, washed, and analyzed for fluorescence intensity, demonstrating engulfment of the particles regardless of genotype or DPI treatment.

Supplemental Figure 6

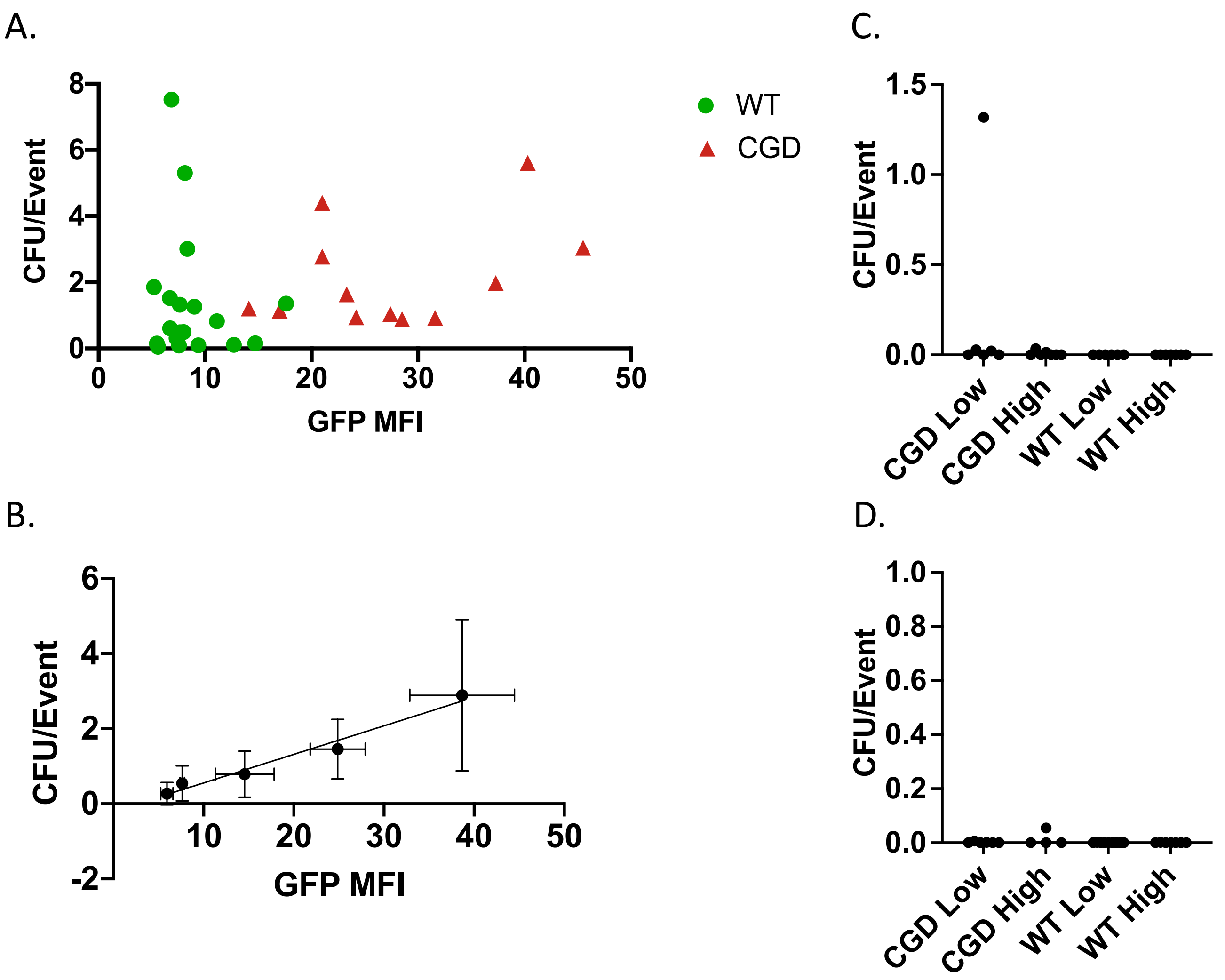

Supplemental Figure 6: BAL neutrophil fluorescent properties relationship with CFU burden follow a similar trend as in vitro infected HB8 neutrophils. (A) Correlation between sorted BAL cell lysates demonstrates a relationship between CFU intracellular burden and GFP MFI for WT and CGD neutrophils. (B) To assess the relationship between GFP MFI and intracellular CFUs regardless of genotype, we binned the data from (A) into five groups based on their GFP MFI rank. (C) ATTO647N+GFP- BAL neutrophils and (D) ATTO647N-GFP- BAL neutrophils do not contain viable *S. aureus.*
